# Editorial: The Effects of Food Processing on Food Components and Their Health Functions

**DOI:** 10.3389/fnut.2022.837956

**Published:** 2022-02-15

**Authors:** Jinkai Zheng, Hang Xiao

**Affiliations:** ^1^Institute of Food Science and Technology, Chinese Academy of Agricultural Sciences, Beijing, China; ^2^Department of Food Science, University of Massachusetts, Amherst, MA, United States

**Keywords:** food processing, nutrients, chemical structure, health functions, mechanism

Food processing plays an important role in our daily diet. It includes a wide variety of treatments on food materials such as mechanical treatment, heating, cooling, drying, high pressure, acid and alkaline treatments, fermentation and more. These processes can significantly influence the composition and structures (chemical structures and multiple-scale structures) of food components and their interactions with one another, thereby significantly affecting their impact on health (1, 2). Food processing could involve in every steps from harvest to the final food products (processed, unprocessed and ultra-processed) we consume.

During food processing, new compounds can be generated that may have health functions (beneficial or harmful). For example, changes induced by heat treatments can produce significant alterations in chemical composition of the food products, affecting palatability, digestibility and bioavailability (3). Both harmful compounds (e.g., acrylamide, HMF, heterocyclic amines) and beneficial compounds (e.g., AGE inhibitors, antioxidant melanoidins) can be produced depending on food formulation and processing conditions (4). Nutrients and bioactive compounds can also be modified in a way that impacts their effects on nutritional status and health: for instance, during the drying of citrus fruits polymethoxyflavones (PMFs) can be transformed to hydroxylated PMFs with more potent bioactivities than the parent compounds (e.g., antioxidative, anti-inflammatory and anti-cancer effects), enhancing the health value of citrus products (5). It is therefore of special significance to elucidate the effects of different types of food processing on food components and the subsequent impact on their health functions.

This volume contains a collection of manuscripts on the effects of food processing on food components and their health functions. Sixteen manuscripts are included: 15 original research articles and one review. They show that various food processing methods (e.g., heat treatment, acid treatment, high-pressure homogenization (HPH), fermentation, frying, emulsification, sun-drying, extraction, and separation methods) significantly influence the chemical structures and functional properties of components in various foods, including skim milk, orange juice, black garlic, soymilk, mango chips, citrus oils, Ganpu tea, deer oil, foxtail millet whole grain flour, pumpkin peptides, gluten epitopes, chrysanthemum, and finger citron.

Eight articles present the effects of processing on food components, including their abundance, chemical structures, and physicochemical properties. Li, Zhao et al. determined the effect of heat treatment on the properties, structure, and aggregation of skim milk proteins. Treating skim milk at different temperatures induced a decrease in sulfhydryl content and an increase in surface hydrophobicity with a disrupted secondary structure of skim milk proteins containing random coils, β-sheets, and β-turns. These changes facilitate aggregate formation via disulfide bonds and hydrophobic interactions. Yu et al. revealed the correlations between HPH-induced changes in the structure of pectin and the stability of orange juice. Compared with non-homogenized orange juice, HPH significantly altered the pectin structure, including monosaccharide composition, molecular weight, linearity, and branching; cracks and pores of different sizes formed on the pectin surface. With increasing pressure and number of homogenization passes, HPH improved the stability of not-from-concentrate orange juice; this was related to the structural properties of pectin. Ma et al. demonstrated that *Lactobacillus* fermentation significantly influenced the physicochemical properties of black garlic extract broth, including its sensory characteristics and composition (e.g., pH, total acids, amino nitrogen, total polyphenols, total flavonoids, and 5-hydroxymethylfurfural). Gas chromatography–mass spectrometry and liquid chromatography–mass spectrometry analyses showed that the contents of several components with unpleasant baking flavors were reduced, whereas the contents of components with green grass, floral, and fruit aromas were increased. More importantly, the contents of functional components like lactic acid, Gly-Pro-Glu, and sorbose were increased after *Lactobacillus* fermentation. The results demonstrated the potential of probiotic fermentation to improve the quality of black garlic. Yang, Yang et al. determined the effect of soybean cultivars on soymilk fermentation using Kefir C. Moreover, new kefir starters were produced by adding lactic acid bacteria to kefir from three different sources: Kefir C, Chinese kefir (Kefir A), and Caucasus kefir (Kefir B). They showed that the starter culture created by adding specific probiotics to Chinese kefir significantly increased the abundance of isoflavone aglycones. Ayustaningwarno et al. evaluated the effect of ripening stage, frying temperature, and time on the quality of vacuum-fried mango. The fat content in fried ripe mango was higher, while the total ascorbic acid in unripe mango remained higher. Considering the quality parameters, vacuum frying unripe mango at the optimal conditions of 100°C for 20 min was preferred for producing high-quality fruit snacks. Yang, Zhang et al. separated essential oils from four citrus varieties into two fractions by molecular distillation. The composition, physicochemical properties, and antimicrobial activity of each essential oil were then evaluated systematically. The main components with antimicrobial activity were 1-decanol, α-terpineol, geraniol, and linalool. Notably, various components in food have low stability or bioavailability (Liu et al.). The development of processing methods such as emulsification to improve their stability and bioavailability has drawn a lot of attention. Xu et al. investigated the effects of ionic strength, heating, and freeze–thaw treatment on the stability of *Monascus* pigment double emulsions stabilized by polyglycerol polyricinoleate (PGPR) and soybean protein isolate (SPI). The *Monascus* pigment double emulsions with <5 mM CaCl_2_ prevented calcium from destroying the physical stability of the emulsions, while those with more than 10 mM CaCl_2_ developed creaming. After freeze–thaw treatment, creaming occurred in the *Monascus* pigment double emulsion. However, it was stable upon heat treatment.

This volume also reports the effects of processing on the health functions of foods that were induced by the changes in their components. The extraction of components from food materials is a common processing procedure. Xia et al. demonstrated that an aqueous enzymatic extract of deer oil not only had a high extraction yield but also had little effect on the contents of active ingredients, especially unsaturated fatty acids, compared with conventional boiling extraction. These components might prevent stomach injury by reducing oxidative stress and inflammation. Hot-water extracts of *Chrysanthemum morifolium* cv. Fubaiju protected against oxidative damage in ARPE-19 cells by activating the PI3K/Akt-mediated Nrf2/HO-1 signaling pathway (Hao et al.). A continuous phase-transition extract of flavonoids from finger citron showed a strong antioxidative activity, as demonstrated by its strong 2,2-diphenyl-1-picrylhydrazyl and 2,2-azino-bis(3-ethyl-benzothiazoline-6-sulfonic acid) diammonium salt radical scavenging activities and oxygen radical absorbance capacity. It also increased the mean and maximum lifespans of *Caenorhabditis elegans* (Luo et al.). A water extract of *Potentilla discolor* improved hepatic glucose homeostasis by regulating gluconeogenesis and glycogen synthesis in mice on a high-fat diet and streptozotocin-induced type 2 diabetic mice (Li, Chang et al.). In addition to extraction, other types of processing were also investigated. Xiao et al. systematically identified and analyzed 104 water-soluble compounds in Ganpu tea and their variation during the sun-drying processing. They showed that the generation of pigments and gallic acid coincided with a sharp decrease in catechin content and significant increases in the alkaloid and flavonoid contents. The conversion of these components helped improve the sensory attributes of Ganpu tea and might be responsible for its unique flavor. Moreover, the components formed during the sun-drying of Ganpu tea had antidepressant effects *in vivo*. Zhang et al. demonstrated that fermentation and germination processing improved the protective effects of foxtail millet whole grain against dextran sulfate sodium-induced acute ulcerative colitis and gut microbiota dysbiosis in C57BL/6 mice. In addition, mice on a diet of fermented–germinated foxtail millet flour had the lowest plasma interleukin-6 levels and claudin2 expression levels in the colon, indicating reduced systemic inflammation and improved gut barrier function. Lu et al. prepared pumpkin seed protein hydrolysate (PSPH) using enzymatic hydrolysis with bromelain, papain, flavourzyme, alcalase, and pepsin. They found that peptides hydrolyzed by papain had the largest average molecular weight, smallest particle size, greatest hydrophobicity, and greatest zinc-binding capacity. Zinc had better gastrointestinal stability in PSPH chelates than as its salt. PSPH-Zn had a higher zinc-binding capacity and better stability. Gao et al. simulated the changes in solution structures of various gluten epitopes at different pH and temperatures to mimic fermentation and baking/cooking processes, and they investigated the binding of differently processed gluten epitopes to human leukocyte antigen (HLA)-DQ2. This revealed that heating and pH change during fermentation affect the solution structure of gluten epitope. However, the binding of differently treated gluten epitope peptide to HLA-DQ2 mainly depended on its primary amino acid sequence, especially the acidic amino acid residues that play a pivotal role in their recognition by HLA-DQ2.

In summary, this collection emphasizes the important influence of food processing on food components and their health effects. This topic is gaining increasing attention. However, some areas are still poorly investigated, such as the impact of processing on the color, flavor, and texture of foods, the effects of chemical structure changes on individual nutrient bioavailability, and their health-promoting effects. Such information would provide critical scientific guidance for establishing optimal processing methods and techniques beneficial to health and is critically important for the production of the next generation of innovative healthy foods.

## Author Contributions

JZ and HX prepared, checked and revised the manuscript, and approved the submitted version. All authors contributed to the article and approved the submitted version.

## Funding

The authors would like to acknowledge the financial support provided by National Natural Science Foundation of China (Nos. 32072181) and Nestle R&D Ltd. The funder was not involved in the study design, collection, analysis, interpretation of data, the writing of this article or the decision to submit it for publication.

## Conflict of Interest

The authors declare that the research was conducted in the absence of any commercial or financial relationships that could be construed as a potential conflict of interest.

## Publisher's Note

All claims expressed in this article are solely those of the authors and do not necessarily represent those of their affiliated organizations, or those of the publisher, the editors and the reviewers. Any product that may be evaluated in this article, or claim that may be made by its manufacturer, is not guaranteed or endorsed by the publisher.

## References

[1] Cui JF Zhao CY Feng LP Han YH Du HJ Xiao H. Pectins from fruits: Relationships between extraction methods, structural characteristics, and functional properties. Trends Food Sci Tech. (2021) 110:39–54. 10.1016/j.tifs.2021.01.077

[2] Azeredo HMC Tonon RV McClements DJ. Designing healthier foods: Reducing the content or digestibility of key nutrients. Trends Food Sci Tech. (2021) 118:459–470. 10.1016/j.tifs.2021.10.02327855881

[3] Zailani MA Kamilah H Husaini A Awang Seruji AZR Sarbini SR. Functional and digestibility properties of sago (Metroxylon sagu) starch modified by microwave heat treatment. Food Hydrocolloid. (2022) 122:107042. 10.1016/j.foodhyd.2021.107042

[4] Zhang NN Zhou Q Fan DM Xiao J Zhao YL Cheng KW. Novel roles of hydrocolloids in foods: Inhibition of toxic maillard reaction products formation and attenuation of their harmful effects. Trends Food Sci Tech. (2021) 111:706–715. 10.1016/j.tifs.2021.03.020

[5] Karn A Zhao CY Yang FL Cui JF Gao ZL Wang MQ. *In-vivo* biotransformation of citrus functional components and their effects on health. Crit Rev Food Sci. (2021) 61:756–76. 10.1080/10408398.2020.1746234 32255367

